# DHEA-dependent and organ-specific regulation of TNF-α mRNA expression in a murine polymicrobial sepsis and trauma model

**DOI:** 10.1186/cc7963

**Published:** 2009-07-13

**Authors:** Tanja Barkhausen, Frank Hildebrand, Christian Krettek, Martijn van Griensven

**Affiliations:** 1 Department of Trauma Surgery, Hannover Medical School, Carl-Neuberg-Strasse 1, D-30625 Hannover, Germany; 2 Ludwig Boltzmann Institute for Experimental and Clinical Traumatology, AUVA Research Center, Donaueschingenstrasse 13, A-1200 Vienna, Austria

## Abstract

**Introduction:**

Dehydroepiandrosterone (DHEA) improves survival after trauma and sepsis, while mechanisms of action are not yet fully understood. Therefore, we investigated the influence of DHEA on local cytokine expression in a two-hit model.

**Methods:**

Male NMRI mice were subjected to femur fracture/hemorrhagic shock and subsequent sepsis. Sham-operated animals were used as controls. DHEA (25 mg/kg) or vehicle was administered daily. Mortality rate, activity and body temperature were determined daily after sepsis induction. TNF-α, IL-1β and IL-10 mRNA expression pattern were investigated in lung and liver tissue after 48 and 96 hours.

**Results:**

DHEA treatment resulted in a significantly reduced mortality rate and improvements in the clinical status. On cytokine level, only TNF-α was significantly reduced in the cecal ligation and puncture (CLP)-vehicle group in both tissues after 48 hours. This suppression could be restored by DHEA administration. In contrast, after 96 hours, TNF-α was up-regulated in the CLP-vehicle group while remaining moderate by DHEA treatment in liver tissue.

**Conclusions:**

The improved outcome after DHEA treatment and trauma is coherent with restoration of TNF-α in liver and lung after 48 hours and a counter-regulatory attenuation of TNF-α in liver after 96 hours. Thus, DHEA seems to act, time and organ dependent, as a potent modulator of TNF-α expression.

## Introduction

Sepsis and associated diseases such as systemic inflammatory response syndrome and compensatory anti-inflammatory response syndrome are common posttraumatic complications in intensive care units. These patients are at high risk of developing multiple organ dysfunction syndrome with subsequent multiple organ failure. Generally, organ dysfunction occurs in a certain sequence. In most cases, the lung is the first organ to fail [1]. When failure of the respiratory system takes place, it is in high frequency followed by liver failure, which develops around day 7 after severe trauma [1].

The early posttraumatic phase is characterized by the abundant production of cytokines such as TNF-α, IL-1β, and IL-6, while in the later posttraumatic course anti-inflammatory mediators such as IL-10 that causes immunosuppression are shown to be more abundant [2]. TNF-α plasma levels correlate with the severity of sepsis and with patients' outcome [3]. Furthermore, it induces the expression of secondary cytokines, such as IL-6 and IL-10. Previous studies of our group showed that induction of sepsis by cecal ligation and puncture (CLP) leads to a significant increase in the plasma levels of TNF-α, IL-6, and IL-10 [4].

The immune system is significantly influenced by the endocrine system. Sex steroids exhibit immunomodulating effects, indicated by gender differences in the susceptibility to sepsis [5,6] and to complications after hemorrhage [7,8]. Several studies have recently demonstrated that the effects of sex steroids are measurable at the cellular level, for example, by reduced splenocyte proliferation or cytokine release [9,10] and in contrast to high IL-6 and IL-10 released by Kupffer cells [10]. These effects could be induced by either high testosterone and/or low estradiol levels [11,12].

Dehydroepiandrosterone (3β-hydroxy-5-androsten-17-one; DHEA) is the most abundant steroid hormone present in the body [13]. Produced by the adrenal glands [13], it serves as a precursor for sex steroids such as estradiol and testosterone [14]. As recently shown, DHEA reduces the mortality rate of mice in CLP models and models of endotoxic shock [14-16]. Previous studies by our group revealed that DHEA effects are partly dependent on IL-6 [4]. Nevertheless, the molecular mechanisms of DHEA action are not completely understood. A functional antagonism of glucocorticoids is suggested, because of the immunoenhancing effect observed after DHEA administration [17]. Furthermore, the effects seem to be partially mediated via the estrogen receptor [18]. In concert with the above mentioned studies, DHEA could be an effective tool in the treatment of sepsis and associated diseases. Because of this, it is of interest to determine molecular mechanisms and functions of DHEA treatment. We therefore investigated the effects of DHEA application in a murine 'two-hit' trauma model consisting of femur fracture/hemorrhage and subsequent sepsis. Special focus of the study was the cytokine mRNA expression pattern in two organ compartments (liver and lung) 48 and 96 hours after sepsis induction. We decided to use those time points because organ failure is expected to occur at these points in the time course, as mentioned above.

## Materials and methods

### Animal care

The study was approved by the animal welfare committee of the state of lower Saxony (Germany). Eighty male NMRI-mice (Charles River, Germany) weighing 20 ± 3 g were used for the study. All animals were handled at room temperature for 14 days before treatment. Throughout the study period, pelleted mouse chow and water were available *ad libitum*. The lighting was maintained on a 12-hour light-dark cycle. Analgesic treatment was performed in all animals (200 mg/kg metamizol-sodium (Novalgin^®^, Hoechst, Unterschleißheim, Germany)) throughout the study.

All surgical procedures were performed after deeply anaesthetizing the animals with ketamine (Ketanest^®^, Pfizer, Berlin, Germany) 100 mg/kg and xylazine (Rompun^®^, Bayer, Leverkusen, Germany) 16 mg/kg. The mice were warmed to 36°C using infrared warming lamps after having finished the surgical procedures. All mice received twice daily subcutaneous injections of 1 ml 0.9% sterile saline for fluid replacement.

### Group distribution and experimental procedures

Four different groups were included in the experimental design (Table 1). The experimental design encloses a two-hit model. The first hit consisted of a closed femur fracture followed by volume-controlled hemorrhagic shock. The standardized femur fracture was induced in both groups using a blunt guillotine device with a weight of 500 g. This resulted in an A-type femoral fracture combined with a moderate soft tissue injury. Two hours later, a hemorrhagic shock was induced by withdrawing 60% of the total blood volume (calculated through the body weight of the animals) via an orbital puncture. Resuscitation using sterile ringer's lactate was performed with four times the shed blood volume in the tail vein after one hour. This means that every animal received an individual resuscitation regime.

**Table 1:** Group distribution

Group	Treatment	Medication	48 hours	96 hours
Sham-vehicle	Femur fracture Hemorrhage Laparotomy	Vehicle	n = 6	n = 6
				
Sham-DHEA	Femur fracture Hemorrhage Laparotomy	DHEA	n = 6	n = 6
				
CLP-vehicle	Femur fracture Hemorrhage CLP	Vehicle	n = 13	n = 19
				
CLP-DHEA	Femur fracture Hemorrhage CLP	DHEA	n = 8	n = 16

CLP = cecal ligation and puncture; DHEA = dehydroepiandrosterone; vehicle = saline containing 0.1% ethanol.

DHEA (25 mg/kg) or vehicle administration was performed subcutaneously once daily until the end of the experiment. In the CLP groups, the second hit was presented by a sepsis induction two days after the first hit (Table 1). As a control, a sham operation with only a laparotomy was performed (Table 1). CLP was performed as previously described [4,19]. Briefly, the cecum was exposed through a midline laparotomy and two unilateral punctures using a 21 gauge needle were performed. Protrusion of the contents of the cecum assured the presence of bacteria in the peritoneum. The abdomen was closed with double layer sutures. All animals were clinically observed and all data obtained until 48 and 96 hours after CLP or laparotomy. We decided to choose time points 48 and 96 hours in this study because organ failure often occurs between these points of time. Lung failure takes place about four days after an insult (which is equivalent to 48 hours following CLP in this study), while liver failure occurs two to three days later.

### Activity score

For quantification of the activity as a measure of the clinical status, a scoring system was used. It differentiates the spontaneous activity, the response to exogenous stimuli, and the amount of spontaneous food intake. The score diverges from 1 to 6 with 6 being very active and gradually decreases to 1 being lethargic (Table 2). The scoring for all mice was independently performed in a blinded fashion by two of the authors (TB and MG). Both observers scored each mouse. The score of each individual mouse consisted of the mean of both values.

**Table 2:** Activity score

Level	Quality	Characteristics of behaviour
**6**	Very active	Strong, curious, fast motions
**5**	Active	Curious, fast, sporadic activity breaks
**4**	Reduced active	Attentive, frequent activity breaks
**3**	Quiet	Disinterested on environment, rare activity, sleepy
**2**	Lethargic	No activity, persist in one position, no food uptake
**1**	Moribund	No activity, reduced vital functions, death is expected

### Body temperature

Body temperature monitoring started at first hit and was performed daily until the end of the observation period. Body temperature was determined with a rectal thermometer (Baxter, UK).

### Body weight

Body weight monitoring started at first hit and was performed daily until the end of the observation period.

### Administration of DHEA

The dosage of DHEA used differs in literature as reviewed in Svec and Porter [20]. The optimal range of dosages used in mice amounts to 25 mg/kg/day. It was reported by Danenberg and colleagues that the mortality due to lipopolysaccharide (LPS) reduced in DHEA dosages between 25 and 100 mg/kg [20]. Therefore, a dosage of 25 mg/kg DHEA was used in this study. DHEA (Sigma-Aldrich GmbH, Deisenhofen, Germany) was dissolved in 70% ethanol. Once daily, 25 mg/kg was injected subcutaneously after the stock solution was diluted in saline. The final concentration of ethanol amounted to 0.1%. This is important as ethanol *per se *can modulate immune responses. Animals of the vehicle group received a once daily injection of saline including 0.1% ethanol.

### Collection of organ samples

For PCR analysis, liver and lung were collected immediately after the mice were euthanized. One lobe of each organ was excised and put into a microfuge tube. The specimens were immediately snap-frozen in liquid nitrogen and stored at -80°C until further processing.

### RNA purification and quantification

For RNA quantification, the frozen organ samples were homogenized in TRIZOL^® ^reagent (Invitrogen, Carlsbad, CA, USA) using an ultraturrax (IKA Labortechnik, Staufen, Germany). The purification was performed as recommended by the TRIZOL protocol. For each sample, 2 μg of purified RNA were reversely transcribed into cDNA by Moloney Murine Leukemia Virus Reverse Transcriptase (Invitrogen, Carlsbad, CA, USA) using oligo(dT)_12–18 _primer (Invitrogen, Carlsbad, CA, USA). Cytokine transcription was detected by semi-quantitative PCR using specific primer pairs for murine TNF-α, IL-1β and IL-10 (Table 3). The amount of the specific PCR product was quantified densitometrically on an agarose gel. Values were normalized by calculating the quotient of amount of cytokine mRNA against the amount of the housekeeping gene glycerealdehyde-3-phosphate dehydrogenase (GAPDH).

**Table 3:** Primer sequences and length of PCR products for TNF-α, IL-1β, IL-10 and GAPDH

Product	Forward primer	Reverse primer	Product size (bp)
TNF-α	ccaagggagagtggtcaggt	ggcaacaaggtagagaggc	317
IL-1β	atcactcattgtggctgtgg	gtcgttgcttggttctcct	322
IL-10	tgctatgctgcctgctctta	gctccactgccttgctctta	405
GAPDH	accacagtccatgccatcac	tccaccaccctgttgctgta	452

### Statistics

Statistical analysis was performed using a standard software application (SPSS Inc., Chicago, IL, USA). Comparisons between groups were performed using one-way analysis of variances (ANOVA) and a *post-hoc *Tukey test. Survival differences were compared using a chi-squared test. To calculate significant differences in cytokine mRNA expression, one-way ANOVA and student's t-test were used. Probability values less then 0.05 were considered statistically significant. The data are expressed as mean ± standard error of the mean.

## Results

### Clinical status and survival

**Figure 1:**
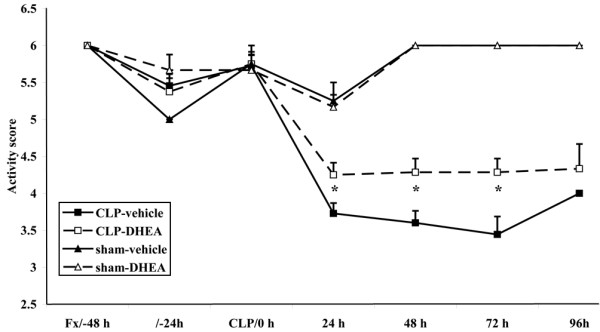
Activity score. The activity score ranges from 1 to 6, with 1 being lethargic and 6 being very active. * *P *≤ 0.05 (comparison of CLP-vehicle and CLP-DHEA). The data are expressed as mean ± standard error of the mean. Black square = CLP-vehicle; white square = CLP-DHEA; black triangle = Sham-vehicle; white triangle = Sham-DHEA. CLP = cecal ligation and puncture; DHEA = dehydroepiandrosterone.

The activity score of mice in sham-operated groups was normal with slight decreases of activity 24 and 72 hours after sham operation. In contrast, mice that underwent CLP showed reduced activity from 24 hours after CLP in comparison to the sham-operated animals (Figure 1). A significant reduction of activity in the CLP-vehicle compared with the CLP-DHEA group could be observed 24, 48 and 72 hours after surgery (*P *< 0.05; Figure 1).

**Figure 2:**
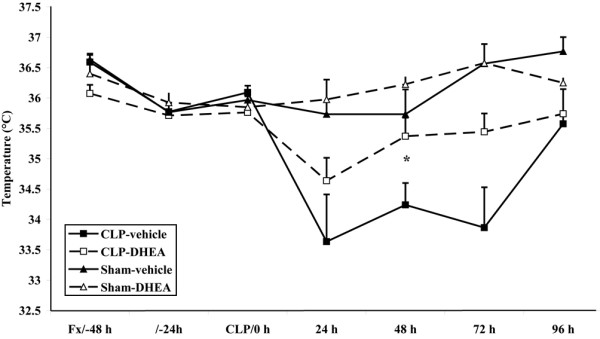
Body temperature. Body temperature (°C) was determined rectally with a thermometer. * *P *≤ 0.05(comparison of CLP-vehicle and CLP-DHEA). The data are expressed as mean ± standard error of the mean. Black square = CLP-vehicle; white square = CLP-DHEA; black triangle = Sham-vehicle; white triangle = Sham-DHEA. CLP = cecal ligation and puncture; DHEA = dehydroepiandrosterone.

Similar to the results of the activity score, the rectal temperature of the CLP animals receiving DHEA treatment was less decreased compared with the CLP-vehicle-treated animals from 24 until 72 hours, with a significantly higher temperature after 48 hours (CLP-vehicle 34.2 ± 1.1°C, CLP-DHEA 35.4 ± 0.7°C; *P *= 0.04; Figure 2). Sham-operated animals showed higher body temperatures than the sepsis groups after treatment.

**Figure 3:**
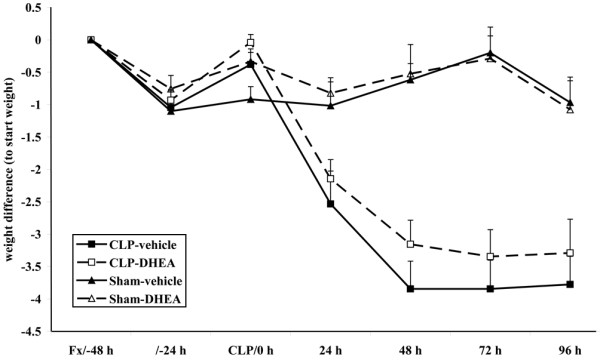
Body weight was determined once daily. The data are expressed as mean ± standard error of the mean. Black square = CLP-vehicle; white square = CLP-DHEA; black triangle = Sham-vehicle; white triangle = Sham-DHEA. CLP = cecal ligation and puncture; DHEA = dehydroepiandrosterone.

We determined differences in body weight throughout the study. However, loss of body weight did not significantly differ between both CLP groups (Figure 3).

**Figure 4:**
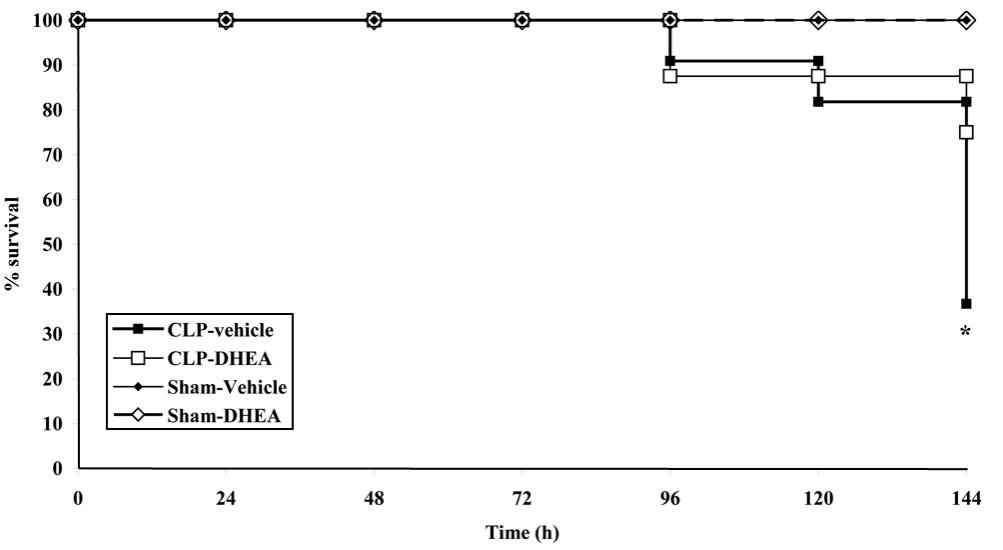
Survival rate. Survival rate (%) of the subgroup that was observed until 96 hours after sepsis onset. Femur fracture/hemorrhage was performed at day 0, sepsis was induced at day 2. Mortality is significantly reduced in the DHEA treated group compared with the vehicle group (* *P *≤ 0.05 using a chi squared test). Black square = CLP-vehicle; white square = CLP-DHEA; black triangle = Sham-vehicle; white triangle = Sham-DHEA. CLP = cecal ligation and puncture; DHEA = dehydroepiandrosterone.

In the sham-operated groups, all animals survived the procedure, with either vehicle or DHEA treatment. In the CLP group with vehicle administration only 36.8% survived the observation period of 96 hours (mortality rate: (12/19) 63.2%). DHEA treatment significantly lowered this mortality to a level of only 25% (4/16; *P *< 0.05; Figure 4).

### TNF-α mRNA expression

**Figure 5:**
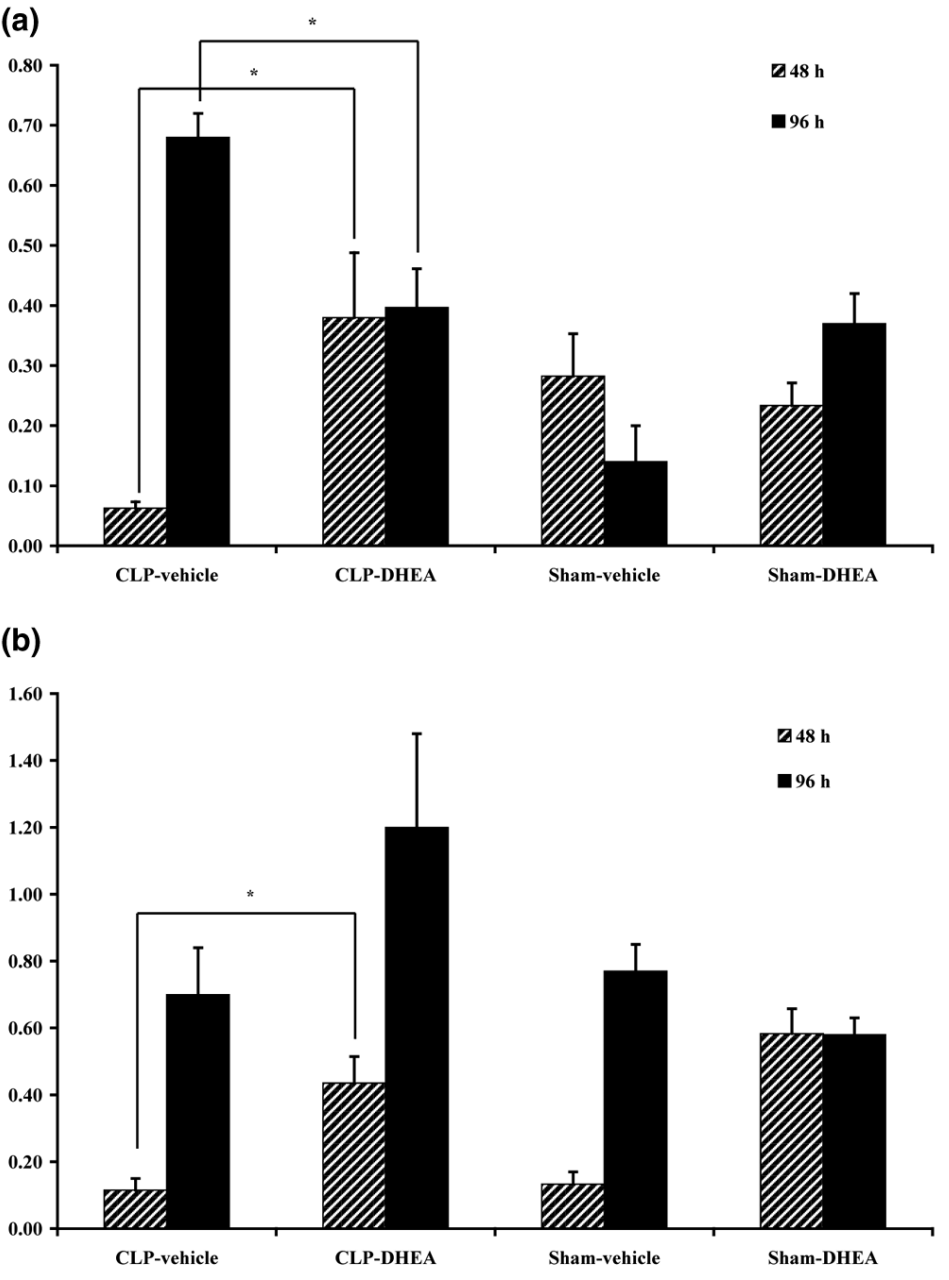
TNF-α expression. **(a) **In liver after 48 and 96 hours. Relative mRNA expression of TNF-α in liver tissue, detected by semi-quantitative RT-PCR 48 and 96 hours after the second hit. The amount of the specific PCR product was quantified densitometrically. The values were normalized by calculating the quotient of the amount of TNF-α mRNA against the amount of mRNA of the housekeeping gene GAPDH. * *P *≤ 0.05. The data are expressed as mean ± standard error of the mean. **(b) **In lung after 48 and 96 hours. Relative mRNA expression of TNF-α in lung tissue, detected by semi-quantitative RT-PCR 48 and 96 hours after the second hit. The amount of the specific PCR product was quantified densitometrically. The values were normalized by calculating the quotient of the amount of TNFα mRNA against the amount of mRNA of the housekeeping gene GAPDH. * *P *≤ 0.05. The data are expressed as mean ± standard error of the mean. CLP = cecal ligation and puncture; DHEA = dehydroepiandrosterone; GAPDH = glyceraldehyde-3-phosphate dehydrogenase.

In liver tissue, TNF-α mRNA expression level was significantly decreased 48 hours after CLP (Figure 5a). Interestingly, DHEA inhibited this repression significantly. Ninety-six hours after CLP, results were inverted in liver, showing an increased expression of TNF-α in the CLP-vehicle group. DHEA caused a return to levels as observed in the sham groups (Figure 5a).

In lung tissue, significant increases in DHEA-treated septic animals versus vehicle-treated septic animals could also be detected at 48 hours (Figure 5b). However, this difference does not seem to be originated in a repression of TNF-α in the vehicle-treated sepsis group, but in a general induction of TNF-α by DHEA as both, sham and sepsis groups, exhibit similar low expression levels while TNF-α is significantly up-regulated in both DHEA groups (sham and sepsis). At 96 hours, we could not determine significant differences between the treatment groups in lung tissue (Figure 5b).

### Plasma TNF-α level

**Figure 6:**
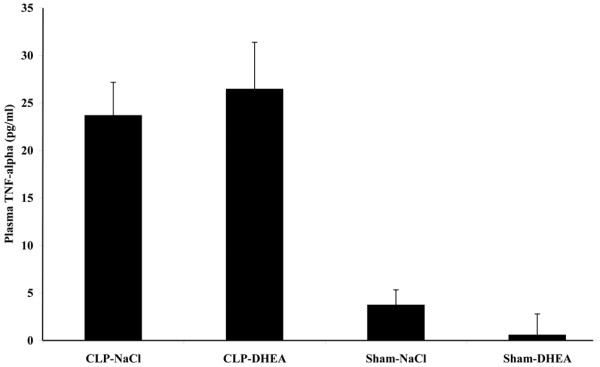
Plasma TNF-α level after 48 hours. Plasma TNF-α level were determined 48 hours after the second hit by ELISA analysis. The data are expressed as mean ± standard error of the mean. CLP = cecal ligation and puncture; DHEA = dehydroepiandrosterone.

**Figure 7:**
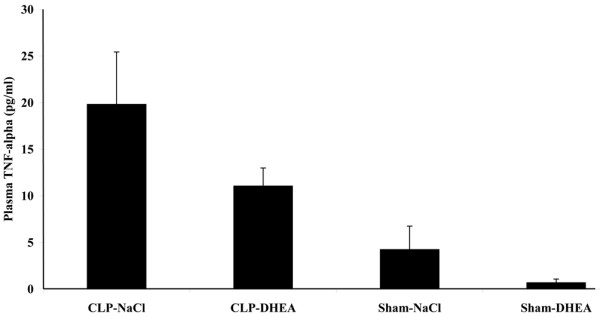
**Plasma TNF-α level after 96 hours**. Plasma TNF-α level were determined 96 hours after the second hit by ELISA analysis. The data are expressed as mean ± standard error of the mean. CLP = cecal ligation and puncture; DHEA = dehydroepiandrosterone.

TNF-α plasma level were already declined 48 and 96 hours after sepsis induction. Levels of the DHEA-treated sepsis group were slightly increased after 48 hours (Figure 6) and controversially slightly reduced after 96 hours (Figure 7) compared with the corresponding vehicle-treated groups. Plasma levels after 48 hours were as followed: CLP-vehicle 23.65 ± 3.51 pg/ml, CLP-DHEA 26.44 ± 4.93 pg/ml, Sham-vehicle 3.71 ± 1.61 pg/ml, Sham-DHEA 0.55 ± 2.44 pg/ml. We determined the following plasma values 96 hours after CLP: CLP-vehicle 19.81 ± 5.62 pg/ml, CLP-DHEA 11.05 ± 1.94 pg/ml, Sham-vehicle 4.23 ± 2.51 pg/ml, Sham-DHEA 0.67 ± 0.4 pg/ml.

### IL-1β expression

IL-1β was expressed in lung as well as in liver tissue. However, IL-1β expression was not significantly altered between vehicle and DHEA treatment in the sepsis groups at any observation point (48 hours and 96 hours) in the tissue types investigated (lung and liver). Liver IL-1β (48 hours): CLP-vehicle 0.55 ± 0.08, CLP-DHEA 0.65 ± 0.06, Sham-vehicle 0.5 ± 0.11, Sham-DHEA 0.53 ± 0.11; Liver IL-1β (96 hours): CLP-vehicle 0.71 ± 0.31, CLP-DHEA 0.96 ± 0.12, Sham-vehicle 0.45 ± 0.16, Sham-DHEA 0.54 ± 0.05; Lung IL-1β (48 hours): CLP-vehicle 0.49 ± 0.16, CLP-DHEA 0.83 ± 0.12, Sham-vehicle 0.57 ± 0.07, Sham-DHEA 0.36 ± 0.06; Lung IL-1β (96 hours): CLP-vehicle 0.50 ± 0.05, CLP-DHEA 0.63 ± 0.13, Sham-vehicle 0.38 ± 0.12, Sham-DHEA 0.46 ± 0.04.

### IL-10 expression

IL-10 was expressed in lung as well as in liver tissue. However, IL-10 expression was not significantly altered between vehicle and DHEA treatment in the sepsis groups at any observation point (48 hours and 96 hours) in the tissue types investigated (lung and liver). Liver IL-10 (48 hours): CLP-vehicle 0.13 ± 0.01, CLP-DHEA 0.20 ± 0.05, Sham-vehicle 0.18 ± 0.02, Sham-DHEA 0.29 ± 0.07; Liver IL-10 (96 hours): CLP-vehicle 0.31 ± 0.03, CLP-DHEA 0.42 ± 0.08, Sham-vehicle 0.24 ± 0.01, Sham-DHEA 0.38 ± 0.02; Lung IL-10 (48 hours): CLP-vehicle 0.21 ± 0.00, CLP-DHEA 0.27 ± 0.06, Sham-vehicle 0.26 ± 0.08, Sham-DHEA 0.35 ± 0.08; Lung IL-10 (96 hours): CLP-vehicle 0.27 ± 0.05, CLP-DHEA 0.28 ± 0.08, Sham-vehicle 0.24 ± 0.06, Sham-DHEA 0.26 ± 0.06.

## Discussion

The data obtained in this study demonstrate that DHEA treatment in a multiple-hit trauma model, consisting of femur fracture with concomitant hemorrhage and subsequent sepsis, exerts protective effects with regard to mortality and the clinical state. Animals undergoing DHEA substitution exhibit significantly lower mortality rates than animals receiving vehicle. Improvements in the clinical status are associated with these results. After sepsis induction, activity is markedly restrained and body temperature declines as well. DHEA treatment ameliorates or even prevents those detrimental effects in septic animals. Our data corroborate the salutary effect of DHEA treatment on clinical status and outcome found in several other studies that were carried out in a variety of disease models such as sepsis, trauma, hemorrhage, viral infections, or burn injury [14-16].

Several studies were performed detecting organ-associated cytokine expression at the protein level. In this context, it is well known that the release of cytokines is repressed in certain stages after trauma and sepsis onset [2,21]. The salutary effect of DHEA administration in trauma and sepsis is well known. In most study designs, a restoration of the repressed immune response could be reported for several cell types by increases in cytokine secretion after DHEA treatment [21-23]. The main focus of this study comprised the role of DHEA in specifically regulating cytokine expression at the mRNA level in the posttraumatic/postseptic course. It was of interest to evaluate if the observed differences in protein level after DHEA administration are caused by changes in cytokine transcription activity. Moreover, we investigated mRNA expression levels in two organ compartments (liver and lung) to determine a possible organ specific and thus differential regulation by DHEA.

In this study, we found that DHEA had a direct action on cytokine mRNA expression 48 hours after sepsis induction in both tissue types investigated. Immune reactivity in the later phases after sepsis onset, in particular TNF-α expression, is typically depressed [24]. This fact can also be documented in this study by a reduction in TNF-α mRNA expression. As our results demonstrate, DHEA administration is able to prevent such a transcriptional repression of the immune response. Animals that underwent CLP and additional DHEA medication show significantly higher TNF-α mRNA expression than vehicle-treated animals. Thus, modulation of TNF-α might be a key factor in DHEA action concerning the protective mechanisms. The importance of TNF-α for sepsis onset is supported by a previous study of our group that demonstrated an important role for TNF-receptor 1 in the septic course. In that study, induction of sepsis by CLP resulted in a mortality rate of nearly 100% in TNF-receptor 1 knock-out mice [4]. Besides, it has already been shown that endogenous TNF-α production, as well as therapeutic TNF-α substitution, have beneficial effects during sepsis of different origins [24-26].

Furthermore, we have to point out that transcriptional modulation of TNF-α represents the most pronounced effect of DHEA in this investigation. It is well known from the literature that DHEA administration influences immune responses, in particular cytokine production, in several animal models [15,22,27]. *Ex vivo *cell cultures show depressed splenocyte proliferation and reduced secretion of IL-1β, IL-2, IL-3, IL-6, IL-10, IL-12 or IFN-γ, depending on cell type [14,18,28]. It has already been observed by several authors that secretion of a number of cytokines was at least partly restored by DHEA treatment [14,18,21,22,28]. Our results are partly congruent with existing literature and go along with the current opinion of a DHEA-dependent restoration of immune suppression after trauma and sepsis. But in contrast to other studies dealing with protein levels, IL-1β and IL-10 mRNA expression levels were not influenced by DHEA in any tissue type investigated in this study. Therefore, we suppose a different time course and/or a differential regulation by DHEA for these cytokines.

In contrast to the results obtained at 48 hours, represented by the suppression of TNF-α in the vehicle-treated sepsis group in both tissue types, expression of this group is strongly up-regulated 96 hours after sepsis induction in liver tissue. However, expression is moderate in all other groups at that time point. This led to the assumption that DHEA suppresses this sepsis-induced increase because levels are normal in animals receiving DHEA after sepsis induction.

Nevertheless, reactions are different in lung tissue. TNF-α levels are not suppressed after 48 hours but remain equal in sepsis and sham groups without medication. In contrast, both groups treated with DHEA exhibited increased TNF-α expression pattern compared with the vehicle groups. After 96 hours, lung tissue exhibits equal levels in all treatment groups without significant peaks. We suggest that organ-specific reactions are responsible for these organ- and time-dependently deviant regulation patterns in the two organ types investigated. This might contribute to a specific sequence in organ failure. As introductorily mentioned, liver and lung are the organs with the most frequent occurrence of organ failure after trauma and sepsis, with lung being the first organ to fail [1]. It is known that early failure of the lung is based on the presence of direct intrapulmonary insults [29], such as ischemia, blunt thoracic injury, and bacterial infection. Furthermore, the lung provides a major capillary net, which might be responsible for early damages because of high amounts of infiltrating immune cells. Our last measuring point (96 hours) is equivalent to six days after the first insult. Liver failure often occurs seven days after an insult [1], thus an association between the detected peak in liver TNF-α expression and liver failure may be present.

Different tissue-specific effects may be explained by receptor expression patterns, receptor densities, or even different receptor types. However, little evidence exists for DHEA intracellular and plasma membrane receptors in some cell types [30-32]. In addition, evidence has been published that DHEA may act via the estrogen receptor [33]. Thereby, a direct activation of the estrogen receptor β by DHEA has been determined [34].

Plasma levels of TNF-α peak a few hours after a traumatic or septic insult and decline afterwards. At the time points determined in this study (48 hours and 96 hours after sepsis induction), plasma levels have almost fallen to normal values and only slight differences between the groups could be determined. Thus, plasma values seem to react independently of the organ-specific cytokine mRNA expression determined in lung and liver.

We suggest that DHEA normalizes mRNA cytokine levels time dependently, with regard to the immunologic tissue context. As initially mentioned, pro- and anti-inflammatory cytokines influence the expression levels of each other. Thus, high initial TNF-α level may result in an increased production of anti-inflammatory cytokines that in turn suppress subsequent formation of TNF-α [35,36]. Additionally, it has already been shown that DHEA action could be interfered by IGF-I, and that a variety of cytokines and growth factors play a role in the modulation of hormone secretion [37,38]. This could result in time-dependently varying reactions and should be evaluated in further studies.

## Conclusions

In this study, we could demonstrate that DHEA improves outcome in a murine polytrauma model. The beneficial effect of DHEA treatment strongly correlates with the restoration of a normally repressed TNF-α mRNA expression in lung and liver 48 hours after the last impact, followed by an attenuation of TNF-α expression in liver after 96 hours in this model. We conclude that DHEA acts time and organ-dependently by regulating the expression pattern of TNF-α. This modulation might partly mediate the beneficial effect of DHEA administration in this polytrauma setting.

## Key messages

• DHEA improves outcome in a murine polytrauma model.

• DHEA modulates TNF-α mRNA expression organ- and time-dependently.

• Changes in TNF-α mRNA expression may be responsible for the DHEA-specific beneficial effect.

## Abbreviations

ANOVA: analysis of variance; CLP: cecal ligation and puncture; DHEA: dehydroepiandrosterone; GAPDH: glyceraldehyde-3-phosphate dehydrogenase; IFN: interferon; IL: interleukin; LPS: lipopolysaccharide; PCR: polymerase chain reaction; TNF: tumour necrosis factor.

## Competing interests

The authors declare that they have no competing interests.

## Authors' contributions

TB made substantial contributions to the data interpretation, performed the experiments statistical analysis and drafted the manuscript. FH and CK participated in the interpretation of data. MG carried out the design of the study, scored the activity of mice and contributed to the interpretation of data.
